# A Neural Network With Logical Reasoning Based on Auxiliary Inputs

**DOI:** 10.3389/frobt.2018.00086

**Published:** 2018-07-30

**Authors:** Fang Wan, Chaoyang Song

**Affiliations:** ^1^Ningquan Technology Co. Ltd., Shenzhen, China; ^2^Southern University of Science and Technology, Shenzhen, China

**Keywords:** logic reasoning, neural network, deep learning, robotic grasping, auxiliary input

## Abstract

This paper describes a neural network design using auxiliary inputs, namely the indicators, that act as the *hints* to explain the predicted outcome through logical reasoning, mimicking the human behavior of deductive reasoning. Besides the original network input and output, we add an auxiliary input that reflects the specific logic of the data to formulate a reasoning process for cross-validation. We found that one can design either meaningful indicators, or even meaningless ones, when using such auxiliary inputs, upon which one can use as the basis of reasoning to explain the predicted outputs. As a result, one can formulate different reasonings to explain the predicted results by designing different sets of auxiliary inputs without the loss of trustworthiness of the outcome. This is similar to human explanation process where one can explain the same observation from different perspectives with reasons. We demonstrate our network concept by using the MNIST data with different sets of auxiliary inputs, where a series of design guidelines are concluded. Later, we validated our results by using a set of images taken from a robotic grasping platform. We found that our network enhanced the last 1–2% of the prediction accuracy while eliminating questionable predictions with self-conflicting logics. Future application of our network with auxiliary inputs can be applied to robotic detection problems such as autonomous object grasping, where the logical reasoning can be introduced to optimize robotic learning.

## 1. Introduction

Current artificial neural networks (ANNs) usually focus on the layers of computation between the input and output for a converging prediction using probabilistic data processing (LeCun et al., 2015). Inspired by the neurons in animal brains, such ANNs are found useful in solving problems which were previously difficult to model using rule-based algorithms (Goodfellow et al., 2016). Both human and artificial learning requires a fair amount of data or examples to establish the learning outcomes, but the human learning also involves deductive reasoning where a wide range of *hints*, i.e., pieces of related-information, to enhance our understanding (Evans et al., 1993; Stenning and Van Lambalgen, 2012). One challenge between human understanding and a computerized algorithm is the exact definition of information, which is strictly required for computer programs but only requires deductive reasoning for the human. One example is through the design and use of the dictionary, which uses a restricted list of *high-frequency words* to help the language learners to understand the explanations and usage of more advanced words (Wittrock, 1974). However, to explain these *high-frequency words*, the dictionaries usually require *circular definitions* of words of similar or related meanings connected through grammar and example usage to hint the language learners to understand with reasons instead of direct memorization. In educational research, such interactive use of hints is found to be a useful tool to enhance the students' learning effectiveness (Munoz-Merino et al., 2011).

Research about learning from hints has been discussed and incorporated in training neural networks (Abu-Mostafa, 1990). For example, one can consider the full explanation or mathematical representation of the reasoning process as a particular case of the hint (Abu-Mostafa, 1993). Suddarth and Kergosien (1990) proposed a “rule-injection hint” method, which adds additionally supervised neurons to the network to improve its performance regarding training time and model generalization. This pioneering work was further developed into a subfield of machine learning, namely multi-task learning (MTL). Caruana (1998) presented evidence and results showing that inductive transfer can improve the generalization of neural networks by exploiting the relatedness among multiple tasks. MTL can also be applied to reinforcement learning (Jaderberg et al., 2016). Recent research also explores the use of hints from a teacher network to train student networks that are much thinner with fewer parameters but comparable performances (Romero et al., 2015).

Combining research in hint related education and learning research, a few general rules for hint design could be summarized. For example, a typical design of the hint should not directly explain the intended concept in full, but partially suggest the concept with pieces of indirect but practical information. Moreover, common usage of the hints is through a series of logical deductions exhausting all possible hints in combination with the knowledge learned to arrive at the most likely concept in question, which is similar to human deductive reasoning from known clues. The interactive use of hints in human learning and reasoning can also be observed from games such as flashcard, hangman, and spelling bees, where the hints are creatively used in different ways and complexity for specific tasks using logical reasoning (Wan and Song, 2017b).

Deep learning, as an extension of machine learning, has made great success in the last few years. Various architectures, such as dense neural networks (Carpenter et al., 1992), convolutional neural networks (LeCun et al., 1989, 1998), recurrent neural networks (Williams and Zipser, 1989) and so on, have been designed to solve specific types of problems in areas of computer vision (Krizhevsky et al., 2012; Verschae and Ruiz-del Solar, 2015), speech recognition (Hinton et al., 2012) and natural language processing (Collobert and Weston, 2008). Deep learning models with multiple inputs are also employed to infuse more information into the models. Chollet (2015) demonstrates a model, which receives the headline as the main input and extra data such as the post time of the headline as an auxiliary input, to predict the popularity of a news headline. For another example, recent work by Levine et al. (2018) has demonstrated the design of a stream of image data supplied to the neural network in parallel to the robot pose information in the Cartesian space to learn hand-eye coordination for robotic grasping. However, it remains challenging for these neural networks to be reasonably understood by a human, except for its underlying probabilistic fundamentals (Knight, 2017).

In this paper, we propose the design of a neural network with logical reasoning through the introduction of *hints* as the auxiliary input, namely *the indicators*. A significant difference of our proposed network is a dynamic reasoning process using these hints to cross-validate the predicted results, which eliminates those with conflicting logics based on the hints used. While reducing the inevitable uncertainties in the data, our proposed network can also be explained using the given hints as the logical basis for human understanding. However, such explanation is also constrained by our understanding of the hints. This is similar to the situation in human reasoning where one can formulate different arguments to explain the same observation using different ways of logical deduction. One potential application of such auxiliary input to a neural network and the logical reasoning is in robotic learning tasks where multiple inputs of different sources and magnitudes can be fused into a network of learning.

For all problems dealing with inevitable uncertainties of new input, there should be a new label of *y*_*conflict*_ that raises questions when inconsistent results are obtained. Humans reason in a similar way when we reach a contrary conclusion from a given input of vague clarity or confusing information (Stenning and Van Lambalgen, 2012), one would naturally question the answer rather than give an illogical one. Our research suggests an interactive interpretation and use of the label information as both input (i.e., hints) and output without compromising the learning outcomes. The abstraction of the indicators can potentially play a more critical role in the understanding of the hidden information in the input data. The generation process for the indicators can be a meaningful one, or randomized, depending on the hint designs. When specific underlying patterns are observable in the labels, the introduction of the indicators as the auxiliary input will contribute information that helps the learning process with a possible explanation.

The next section further explores the design variations of the indicators as the auxiliary input for the proposed neural network structure using the MNIST example and a new data set collected from robotic grasping. Section 3 compares the experimental results and discusses the design principles when generating these indicators from the labels. Section 4 discusses a potential application of the proposed neural network with auxiliary inputs in robotic learning for object grasping. Final remarks, limitations and future work are enclosed in the last section, which ends this paper.

## 2. The design of a neural network with hints

The introduction of hints for logical reasoning is the primary differentiation behind our proposed network, which aims at direct or indirect suggestions bridging the input data and the output labels with a logical decision-making process. Since these indicators are suggestions abstracted from the original data, i.e., images and labels, a review of the information embedded in these images and labels become essential. In general, the labeled outcome usually corresponds to a comprehensive and sophisticated question that is hard to answer and therefore cannot be easily modeled using the existing knowledge. Although the final reply to the question may be framed as a simple *Yes* or *No*, one could usually break down the question into further details for more specific inquiry. For example, in the problem of identifying the breed of a cat from a collection of different cat and dog breeds, one can approach the answer by taking two steps: (1) *what is the animal in general* and (2) *what is the specific breed of this animal*. Such operation may have different logical complexities depending on how one strategize to obtain the solution. It is similar to the scenario when people are trying to find solutions to the same problem. Some may follow the existing procedures that are straightforward but difficult in practice, while others might identify particular patterns from the questions and possible answers to formulate a logical process that narrows down the maxim. Our network is similar to the latter one, trying to mimic deductive human intelligence using the hints.

### 2.1. The auxiliary input and new labels

**Figure 1:**
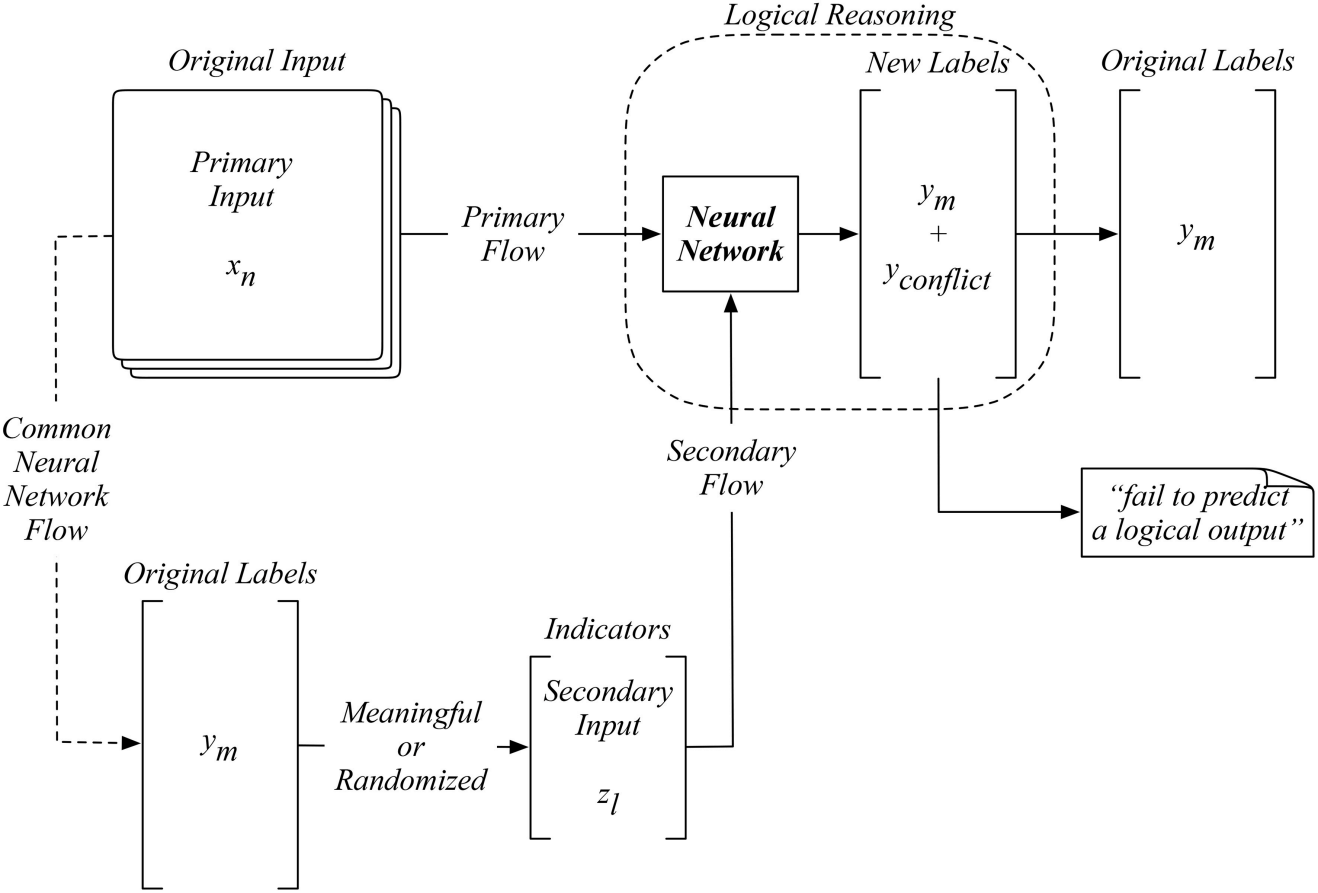
The design of a neural network with an auxiliary input generated from labels to improve learning accuracy.

Before our training starts, we are presented with a set of input data with labeled output as the *prior information*. In our proposed neural network shown in Figure 1, the original input is still used as the primary input ***X*_*N*_** = {*x*_1_, *x*_2_, …, *x*_*N*_}. Also, we generate a set of auxiliary input to the network, namely the indicators, by categorizing the original labels ***Y*_*M*_** = {*y*_1_, *y*_2_, …, *y*_*M*_}. Since the understanding of these labels is not directly linked to the input, we can exploit such *indirect* knowledge that is not presented in the prior data to categorize these output labels, which can be a meaningful process or even a randomized one. The resultant indirect suggestions become a set of indicators ***Z*_*L*_** = {*z*_1_, *z*_2_, …, *z*_*L*_} which usually has a smaller dimension than the original labels (*L*<*M*) as a conceptual abstraction. For logical modeling, any original input *x*_*i*_ shall be led to its original output *y*_*i*_ through the involvement of a particular indicator *z*_*i*_ that suggest this correct computation. When computing this original input *x*_*i*_ with all other indicators, a new label *y*_*conflict*_ becomes necessary to differentiate these illogical outcomes from the logical one. Therefore, we shall have a set of new labeled output ***Y*_*M*+1_** = {*y*_1_, *y*_2_, …, *y*_*M*_, *y*_*conflict*_} when training our network. One can further exploit the concept of hints by designing multi-dimensional or direct indicators. One can also design a new set of restructured labels from the original input data for more advanced logical learning. Both of these scenarios will be explored in the later sections of this paper.

### 2.2. New architecture for logical learning

Our proposed network takes a primary input *x*_*i*_ and an auxiliary one *z*_*i*_ to model a new set of labeled output *y*_*i*_ during training, as shown in Figure 1. This neural network is structurally different by adding a process that establishes a certain logical relationship in the original data. The advantage of our network is the full exploitation of the existing understandings of the problem through the design of meaningful indicators and the establishment of logical reasoning. Later we will also show that the training process can be further exploited by designing randomized indicators to suggest unknown relationships and reduce the data and logic uncertainty.

**Figure 2:**
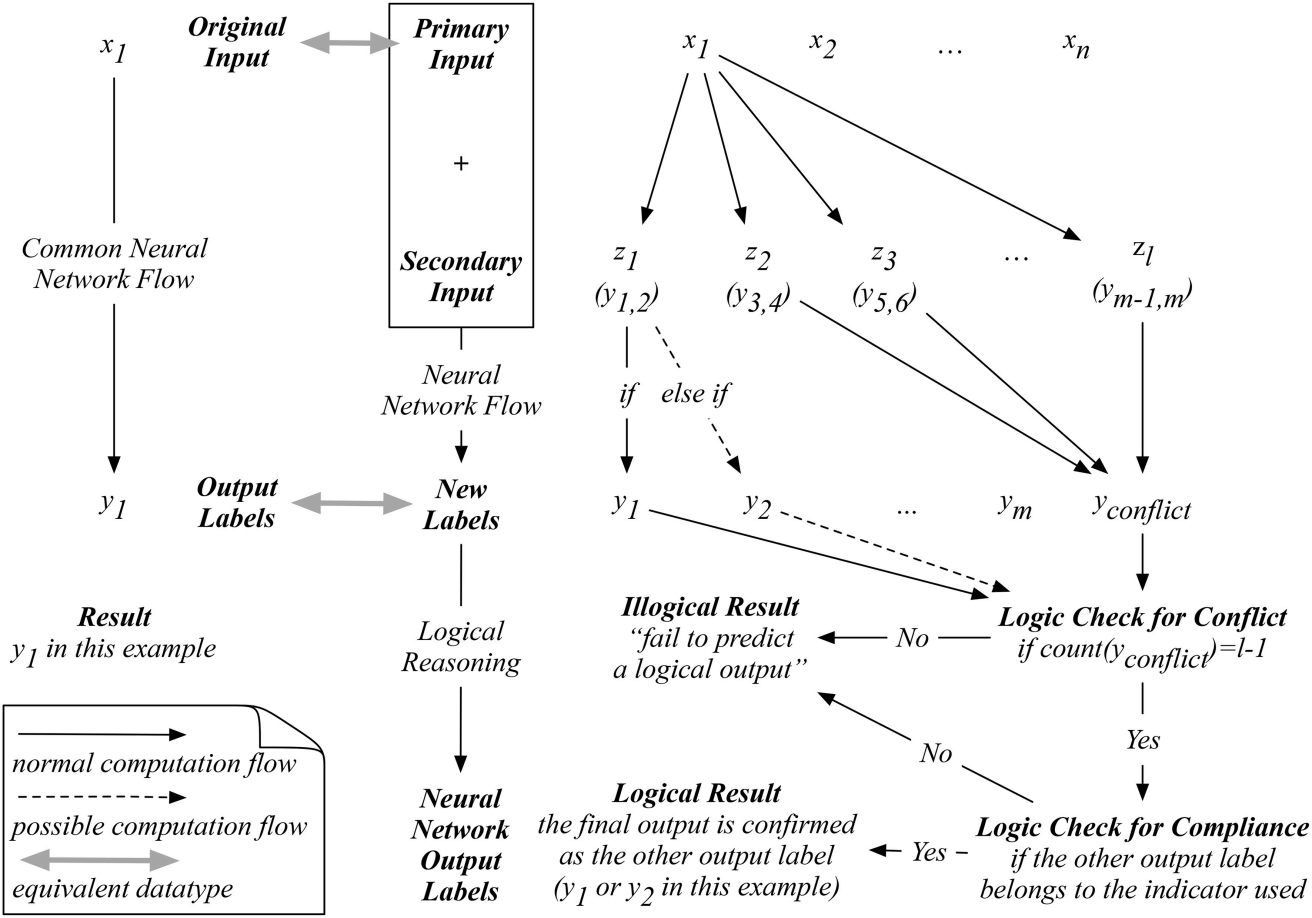
An example of the computation flow in the proposed network with logical reasoning.

Since our auxiliary input is an artificial one, we are only provided with the original input to proceed with our prediction. For example, as shown in Figure 2, when a new input *x*_1_ is presented, our proposed neural network on the right will exhaust all possible indicators in ***Z*_*L*_**. To logically determine a reasonable prediction, we start by counting whether the number of *y*_*conflict*_ predicted equals to *L*−1, which means that only one *non-conflicting* label is predicted after the exhaustion of all possible indicators. If so, the next logical check is to determine if the *one-and-only non-conflicting* label complies with the corresponding indicator used to predict this non-conflicting label. If this one-and-only non-conflicting label passes both logical reasoning tests, then a logical result is obtained by our network. Otherwise, an illogical result will be reached with a statement such as “fail to predict a logical output.” This process differentiates our proposed network from the existing ones, where an output label will always be computed without cross-validating the logic behind.

### 2.3. The adapted MNIST data

**Figure 3:**
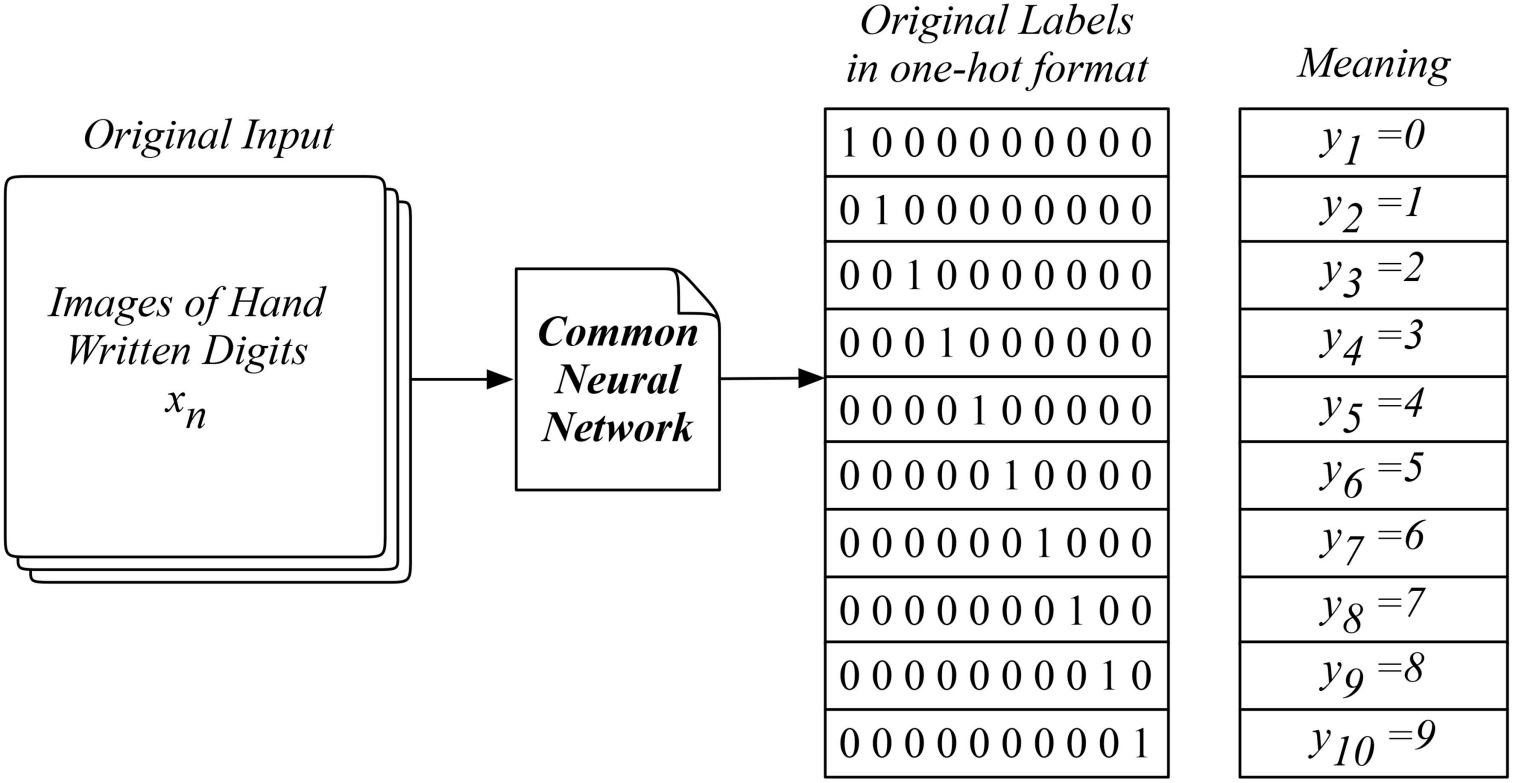
The MNIST data and a common neural network widely used in machine learning.

The MNIST data is an extensive image collection of handwritten digits labeled from zero to nine, consisting of a training set of 60,000 examples and a test set of 10,000 examples, which is widely adopted as a benchmarking dataset for learning research and practice (LeCun et al., 1998). Researchers usually adopt the common neural network which uses the images as the input data ***X*_*N*×28 × 28 × 1_** and the ten digits as the output labels ***Y*_10_** as shown in Figure 3. In practice, the model outcomes are effectively labeled in the one-hot format which corresponds to the ten different digits. These ten digits are treated as ten independent categories disregarding their mathematical meanings and relationships behind. This *direct learning* method is comparable to the way we approach the dictionary for the meaning of a new word. However, it hardly reflects the logical and interactive way we naturally adopt when using words in a language, which requires understanding the logic behind.

**Figure 4:**
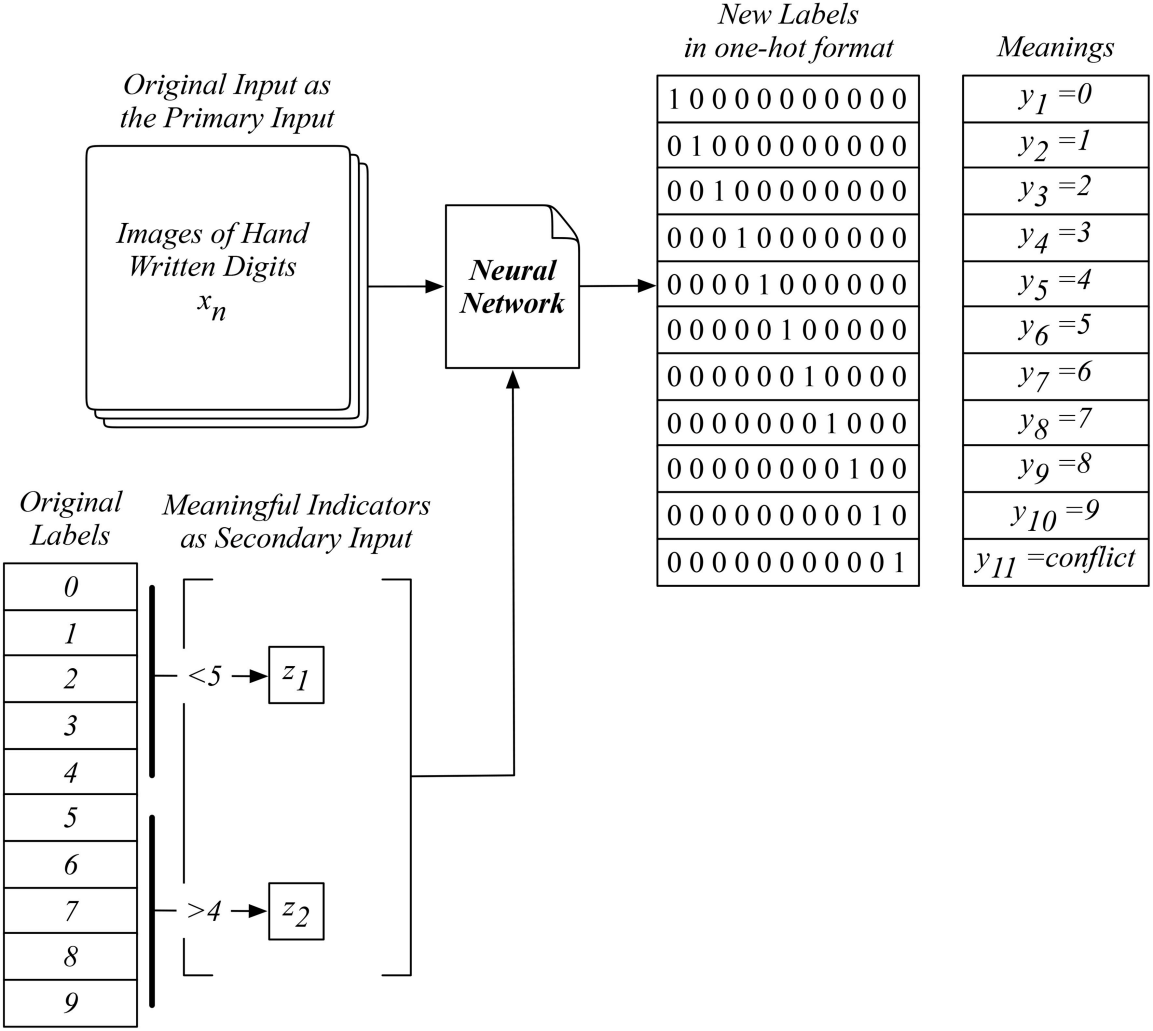
An example of our proposed neural network with auxiliary inputs for training using an adapted MNIST data with two indicators, suggesting original labels smaller than five or not, as the auxiliary input.

The example in Figure 4 shows the training setup with a set of two indicators, with *z*_1_ suggesting the label being smaller than five and *z*_2_ suggesting otherwise. The training of our network involves the combination of the primary input and all possible indicators. For example, with an image of digit zero as the input, we generate the label when this image and indicator *z*_1_ are used, which leads to the correct label of *y*_1_ = 0. In the meanwhile, we also generate another label when this image and the other indicator *z*_2_ are used, which leads to the conflicting output label of *y*_*conflict*_. This process essentially tells our network to learn both sides of the knowledge, including the correct and conflicting ones. The introduction of this new label *y*_*conflict*_ enables our network to capture the logic of this new knowledge during training, which will be used for the logical reasoning when testing our network.

**Figure 5:**
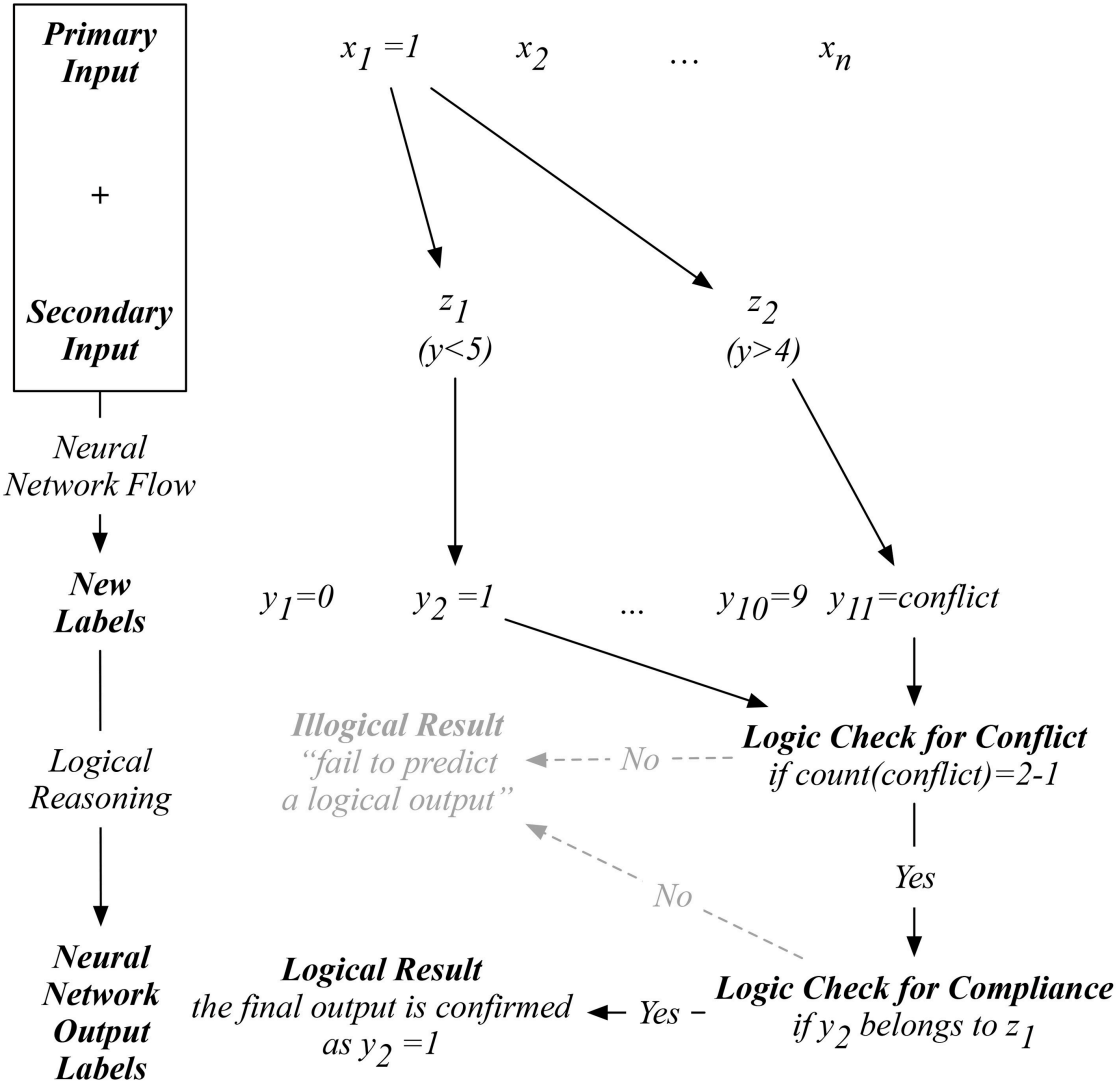
An example of our proposed neural network with auxiliary inputs for a logical output using the MNIST data.

Testing our network involves an exhaustive computation between a given primary input and all possible indicators, and a logical reasoning process trying to make sense out of the predicted results. The example in Figure 5 demonstrates the case when an image *x*_1_ of digit 1 is used for testing. Our network compute *L* = 2 label outputs that correspond to all combinations of *x*_1_ and *z*_1_, and *x*_1_ and *z*_2_ as the input pairs. Then, in the logical reasoning flow, we first check if the total number of *y*_*conflict*_ predicted equals to *L*−1, meaning that only one non-conflicting label is predicted. Then, we further check if this non-conflicting label complies with the corresponding indicator used for its computation. A logical result can only be obtained when a non-conflicting label complies with the indicator used for its computation. If the set of predicted labels fail to pass any of these two logical checks, we can still arrive at an output label by selecting those with lower computation confidence, just like the method used in the common neural networks. However, this will only result in illogical results, which the existing neural network can not detect. An extreme example is when one supplies an entirely irrelevant image to a neural network, like a picture of a cat instead of a hand-written digit. The existing neural network will process it with a labeled output, possibly with very low confidence, asking for manual checking. It corresponds to a statement such as “the most probable recognition of this new image is a digit …”. However, our network can identify all possible labels computed to collectively suggest an illogical output, which corresponds to a statement such as “no logical recognition can be computed,” suggesting something is wrong with this image or the model currently in use for this task based on the knowledge learned.

For demonstration, the original MNIST data has been adapted in various ways in the following section by introducing different designs of the indicators to restructure the neural network. In principle, we can design a few sets of the indicators to suggest the ten digits according to (1) the meaning of the indicators, (2) the total number of indicators, and (3) the number of labels suggested by each indicator.

We first design four sets of indicators marked as 11–14 in Table 1 with mathematical meanings. The ten digits are divided into two categories, including case 11 of smaller than 5 or not (equally divided), case 12 of even number or not (equally divided), case 13 of prime number or not (unequally divided), and case 14 of zero or not (unequally divided). We also randomly generate four sets of indicators marked as 21–24 in Table 1 with different total counts of *L* and size distributions. All sets of indicators in Table 1 provide indirect suggestions to all ten digit labels except for cases 14 and 24. Case 14 contains a direct suggestion of an indicator zero to label zero, and case 24 contains direct suggestions of the ten indicators to the ten digit labels respectively. Both designs of the indicators violate the general design guidelines of hints as defined earlier. More specifically, case 14 represents a partial violation of the hints with a direct suggestion of digit 0 whereas case 24 accounts for a total breach of a specific suggestion of all ten digits. However, whether such violation is a bad design remains an open question, which will be discussed later.

**Table 1:** Experiment designs and prediction results of the auxiliary inputs as the indicators for the MNIST data.

**No**	**Indicator meanings**	**Indicator characteristics**	**Output labels**	**Logical results (%)**	**Logical accuracy (%)**	**Overall accuracy (%)**
11	Smaller than 5	2 indicators	Ten digits	99.48	99.24	98.72
	or not	equally distributed (5, 5)				
12	Even number	2 indicators		99.49	99.21	98.70
	or not	equally distributed (5, 5)				
13	Prime number	2 indicators		99.57	99.13	98.70
	or not	unequally distributed (4, 6)				
14	Zero or not	2 indicators		99.90	99.00	98.90
		unequally distributed (1, 9)				
21	None	2 indicators		99.36	99.17	98.54
		equally distributed (5, 5)				
22	None	2 indicators		99.40	99.32	98.72
		unequally distributed (3, 7)				
23	None	5 indicators		98.79	99.39	98.19
		equally distributed (2 × 5)				
24	None	10 indicators		98.53	99.41	97.95
		equally distributed (1 × 10)				
31	Case 11 + case 12	4 indicators	Ten digits	98.97	99.33	98.31
		combining Cases 11 and 12				
32	Ten digits	10 indicators	Case 11	97.33	99.36	96.71
		equally distributed (1 × 10)				
33	Case 12	2 indicators	Case 11	99.58	99.33	98.91
		equally distributed (5-5)				

Since the indicators should only provide indirect suggestions, we can also design new prediction models as shown in Table 1 by interchanging the labels and the indicators used in our network. One particular design of the indicators is a multi-dimensional indirect suggestion. For example, each digit can be simultaneously identified as smaller than five and an even number. This corresponds to case 31 in Table 1 where a set of two-dimensional indicators carries the meanings of both cases 11 and 12. Another special design is through the exchange of the indicators and labels to formulate a revered prediction, as shown in case 32 in Table 1. A further special design is to hide the exact information of the ten digit by using indirect suggestions in both indicators and labels, such as the case 33 in Table 1. Note that in this case, since we are losing the exact information of the specific digit on the image, we can only do the first layer of the logic check for conflicts but cannot proceed to the second layer of the logic check for compliance.

## 3. Experimental results and discussions

We benchmark our models with the MNIST data using a Convolutional Neural Network that contains three convolutional layers (5 × 5 × 1 × 4 with stride 1, 4 × 4 × 4 × 8 with stride 2, and 4 × 4 × 8 × 12 with stride 2) and two fully connected layers (588 × 200 and 200 × 10). The test accuracy of the benchmark model maximizes at 98.91% after 10,000 training steps with training batch equals to 100. One can further improve the accuracy by referring to Görner (2016) using more advanced techniques such as more layers, batch normalization, dropout and ReLu layers, which are not used in our experiments. We further tested our network using a dataset collected from the DeepClaw robotic grasping platform, which will be explained at the end of this section.

All experiment results are reported in Table 1. The *Logical Results* column refers to the percentage of the 10,000 MNIST testing examples that pass the two layers of logical checks. This self-checking mechanism leads to two prediction accuracies of interest. The *Logical Accuracy* indicate the prediction accuracy of the logical results. The *Overall Accuracy* indicate the prediction accuracy of all results, including the *Illogical* ones with failed classification. Note that in our experiments, it is unlikely for the overall accuracy to be higher than 98.91% in the original network when no logical reasoning is considered.

### 3.1. Any indicator is a good indicator for logical reasoning

As shown in Table 1, all logical results are above 99% prediction accuracy, higher than the original neural network's benchmarking prediction accuracy at 98.91%. We observed statistical significance in the different prediction accuracies between the logical and overall results with a *p*-value of 0.0037 among cases 11–33. Irrespective of the indicator designs, our results suggest enhanced trustworthiness by eliminating the illogical predictions, which is not available in the original neural network. Common neural networks only adopt the direct information presented in the data but ignores the logic behind, which is usually a reasoning process of human thinking instead of memorizing past events using brutal force computation. These logics help to deal with the uncertainties in the future events. The robustness of the results shown in Table 1 demonstrates the effectiveness of the proposed logical learning through a neural network with auxiliary inputs. These indicators are flexible in design to provide indirect (cases 11–13 and 21–23) or direct (cases 14 and 24) suggestions for the labeled outputs. Moreover, the designs and understandings of these indicators are the prior knowledge that the human operators had previously acquired from the given dataset for artificial learning.

### 3.2. Logical complexity positively relates to the confidence of a logical answer

**Figure 6:**
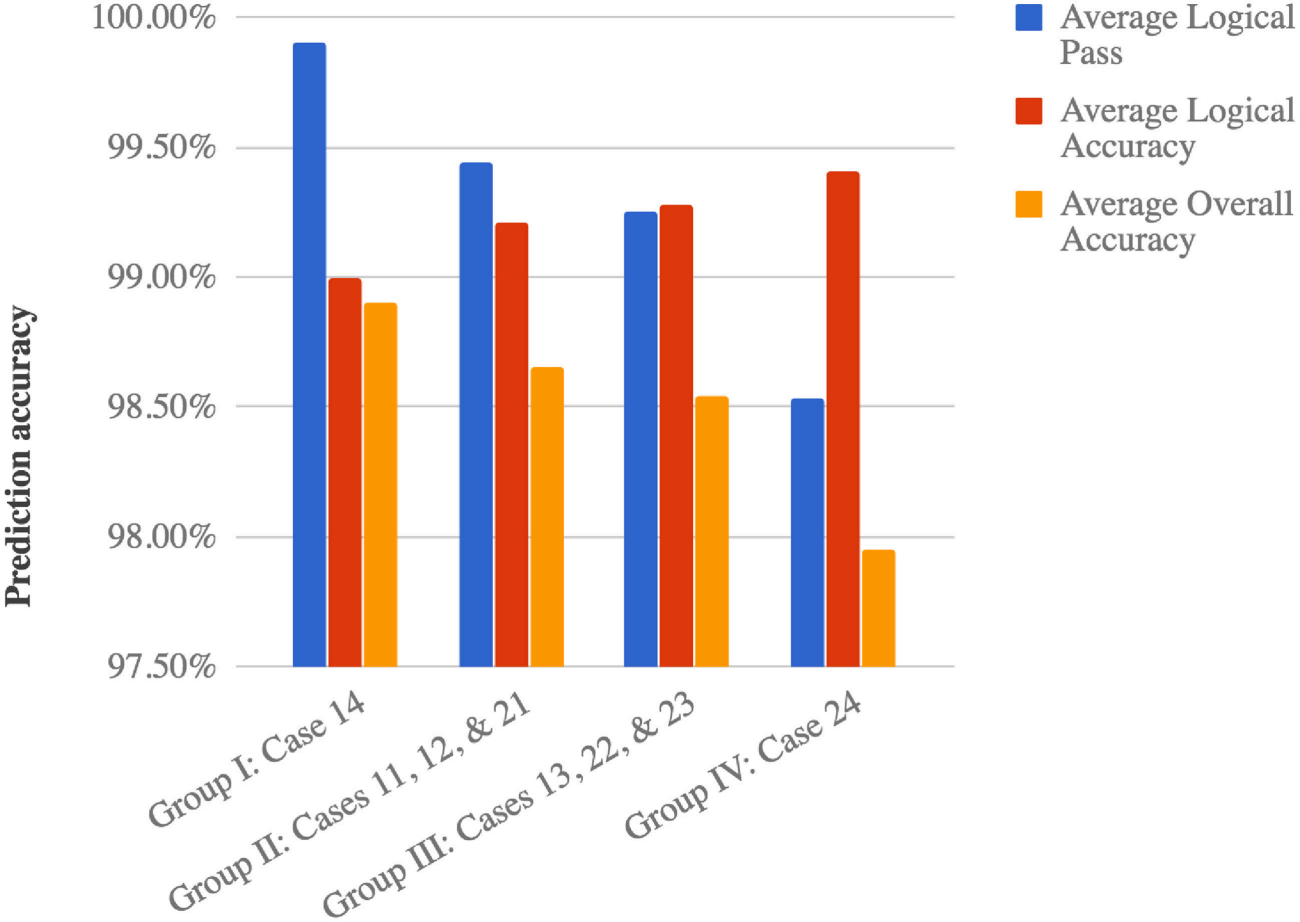
The average results of the four case groups of the normal indicators used.

As shown in Table 1, three levels of concept in cases 1x, 2x, and 3x are used to represent different logical complexities when designing the indicators. Those generated from random partially reflects a certain degree of unknown logic with the highest complexity, whereas the meaningful ones are more straightforward for human understanding. The count of indicators is also an important aspect to reflect the complexity of the logical reasoning in our networks. For example, every added indicator would boost the required training by one fold as we traverse all the indicators. Furthermore, the distribution of each indicator and the labels it suggests also present a statistical influence that correlates to the distribution of the input data. In general, we can divide these eight experiments into four groups according to their logic complexities. We observe a growing trend of logical accuracy as the logic complexity increases, as shown in Figure 6.

- Case 14 reflects the group with the least logical complexity, which shares the most similarity to the original MNIST data with the lowest logical accuracy of 99.00%, but still higher than the original network. This is effectively a direct indication for images of zero and a reduced MNIST prediction without the zeros. Such simplicity on logic leads to the highest percentage of passing the logical checks at 99.90%.
- The second group contains cases 11, 12, and 21 with slightly more complex logical relationships, which are either meaningful or randomized indicators, and all have a set of two indicators suggesting an equal number of labels. An average logical accuracy of 99.21% is reached with the percentage of passing the logical checks reduced slightly to 99.44%.
- The third group consisting of cases 13, 22, and 23 presents more complexity in all three aspects of indicative meaning, indicator count, and size distribution. For example, both cases 13 and 23 have unequal size distributions comparing to Group 2. Moreover, the total count of indicators increases to five even though the size distribution stays equal. This group achieves an increased average logical accuracy of 99.28% with a 99.25% passing rate of the logical checks.
- The fourth group only contains case 24, which is the most complex logic with ten indicators of direct suggestions for training. It produces the highest logical accuracy of 99.41%. The side effect of such restrict logic is that the passing rate of the logical checks only 98.53%, the lowest in our experiments.

### 3.3. Logical result is at the cost of overall accuracy

The logical result offers us with a prediction with enhanced trustworthiness when dealing with the unknown uncertainties of a question, i.e., a new input data. The prediction accuracy of the overall result also provides us with a way to compare our network with the original one. We also plotted the trend of overall prediction accuracies of the four experiment mentioned above in Figure 6. A decreasing trend can be observed as the accuracy of the logical results increases. The percentage of logical pass also shows a strong influence on the overall prediction accuracy in Figure 6. This is possibly caused by the rapid decrease in the logical pass, which is about three times faster than the increase in logical accuracy. The following equation can be used to reflect such relationship.

(1) $$\begin{array}{r} OverallAccuracy=LogicalAccuracy\times LogicalPass \end{array}$$

### 3.4. Meaningful indicators are not that good, and direct suggestions are not that bad

Another observation is about the design of the indicators in their meanings and suggestive strength. Among the cases 1x with meaningful indicator designs, the differences in the prediction accuracies between logical and overall results exhibited statistical significance with a *p*-value of 0.0290. Among cases 2x with directly suggestive indicators, we observed a similar statistical significance with an even smaller *p*-value of 0.0198. Comparing cases 11, 12, and 21, we see that the meanings of the indicators only present a slight increase in the prediction accuracy of the logical results from 99.17% to 99.21–99.24%. One probable explanation is that the computers do not perceive such mathematical relationships much differently from the randomized ones. This is different from the human learning, which requires the meaningful perception of the concepts to better understand. This partially reflects the underlying difference between the humans and the machines, where such slight increase in the understanding makes a significant difference in the autonomy of minds.

While designing these indicators, we intentionally tried to mimic the human use of hints indirectly as introduced at the beginning of this paper. Among the eight normal experiments, only cases 14 and 24 involves direct 1-to-1 suggestions between the indicators and the labels. These two cases outperformed the rest ones in logical accuracy. All eight experiments present an average logical accuracy at 99.23% (0.0014 standard deviations) and an average overall accuracy of 98.55% (0.0032 standard deviations). From our results, it seems challenging to draw a clear distinction between the effects between direct and indirect suggestions.

### 3.5. Special design and use of the indicators

We also list a few special cases in Table 1 to demonstrate the versatile design and use of the indicators in our network, with interesting results reported in Table 1.

Case 31 is a two-dimensional design of the indicators which combines case 11 and case 12. The result shows an increase in the prediction accuracy of the logical result of 99.33%, but a decrease in the passing rate of the logical check to 98.97%. In principle, it is possible to design such multi-dimensional indicators. However, the major difference is more operational instead of numerical. Multi-dimensional indicators require each input to be supplied with multiple indicators instead of one as shown in Figure 1. This design is equivalent to four one-dimensional indicators.

Case 32 is a reversed prediction by exchanging the labels and indicators in case 11. The primary interest of this case is the application of our network when the number of the indicators are larger than the number of labels. In this case, we use ten meaningful indicators to suggest two possible labels given an input data. The passing rate of the logical check is found to be the smallest among all experiments, suggesting the highest logical complexity and resulting in the lowest overall prediction accuracy. However, we did not suffer any significant decrease in logical accuracy, which remains at a relatively high level of 99.36%. This case requires the same amount of computation as case 24 to traverse all indicators. By applying the conclusion of “*meaningful indicators are not that good, and direct suggestions are not that bad”* obtained in the last section, it seems reasonable to introduce a random set of indicators whose size is bigger than that of the output labels to reach a reasonably high logical accuracy. This is especially useful when the cost of data acquisition is high, such as autonomous robotic grasping task, which will be further explored with more details in the next section.

The last case 33 completely buries the explicit information of the ten numerical digits in the data. The overall accuracy is the highest among all normal and special neural networks with auxiliary inputs at 98.91%. It is interesting to notice that this result coincides with the original neural network's prediction accuracy. The result for case 33 suggests that the use of hints may be the most suitable one for indirect suggestions to effectively address unknown uncertainties and find new knowledge that was not previously expressed in the original data. One drawback of case 33 is that the second logic check cannot be performed as we cannot decide if a number smaller than five is an even number or not. Further research is required to dive further into this kind of problem.

### 3.6. Experiment using image data collected from deepclaw

**Figure 7:**
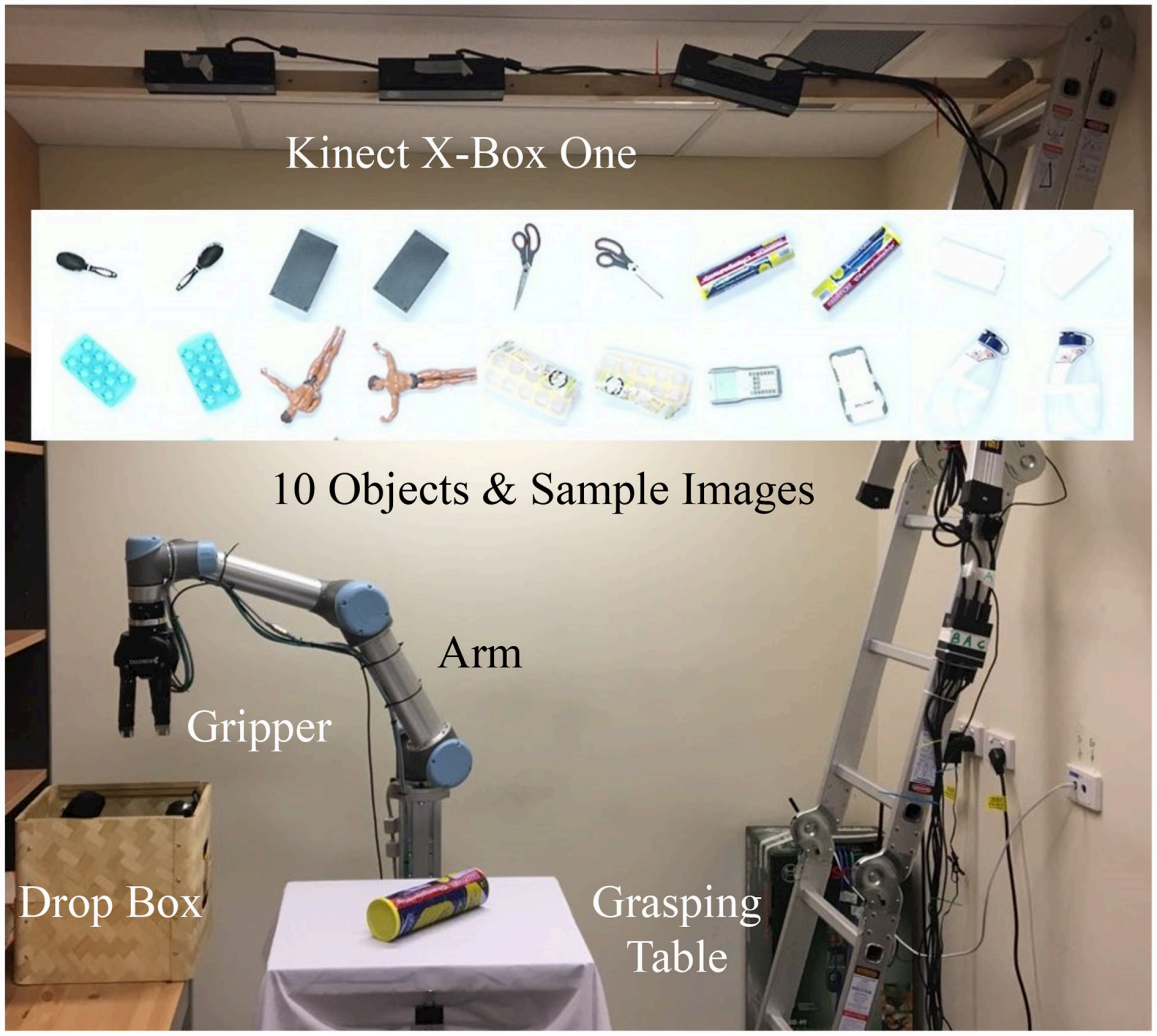
The DeepClaw grasping setup mimicking the arcade claw machine game using a UR5 robot arm, a Robotiq gripper, a Kinect for Xbox One camera at the center, and a PC with NVidia Titan X 12G GPU to pick up objects from the table. Ten daily life objects were used with 2 sample pictures shown in the middle. One thousand grasping attempts were performed on each object in the collected dataset.

We further experimented our network using a dataset of 10,000 images collected by a *DeepClaw* platform as shown in Figure 7. Ten daily life objects were used to perform autonomous grasping tasks to pick up an object and put it back with a random pose to the table. One end of a transparent fish line is tied to the object through a hole in the center of the table while the other end is tied to a weight. Each time the object is released, the weight under the table will pull the object back to the table center at a random pose. Only the images taken by the camera in the center were used, and each object is picked up 1,000 times. Further details of this robotic setup are available on a Github repository (Wan and Song, 2017a).

To test our network, we adopted the same network structure as cases 21 and 23 to design the indicators for the ten objects, which are randomly generated. 9,000 of the images were used for training and the rest 1,000 were used for testing. Each image is labeled in the original data and similar to the MNIST data, the network's goal was to classify them correctly into ten categories. As shown in Table 2, a typical neural network can already classify these ten objects with 98.0% accuracy, which is comparable to the MNIST data. However, in two experiments using our network with 2 or 5 random indicators for these ten objects, we can further improve the accuracy to 98.5 or 99.4% using logical reasoning. Our results demonstrated consistent observations from previous experiments using the MNIST data. The introduction of the indicators further improved the prediction accuracy in both Cases 41 and 42. With more indicators, or more complex indicators, case 42 is observed to outperform case 41 in logical accuracy. This is at the cost of a lower percentage of logical results and overall accuracy, which is caused by the increase in illogical predictions detected by the increased number of indicators. The logical reasoning behind our results is embedded in the design of indicators, which provides a structure of understanding to the results produced by the neural networks. Although we still cannot literately understand the meaning of the results using these randomly generated indicators, our network provides a possible structure of taxonomy through these hints to approach a possible interpretation, which is not available in the previous literature.

**Table 2:** Comparison between neural networks without (Case 40), with 2 random (Case 41), and with 5 random (Case 42) auxiliary inputs using grasping images collected from the DeepClaw platform, where the introduction of indicators differentiate illogical predictions with improved logical accuracies using logical reasoning.

**No**	**Auxiliary indicators**	**Logical results (%)**	**Logical accuracy (%)**	**Overall accuracy (%)**
40	Common network with no indicators	Not applicable	Not applicable	98.0
41	Random 2 indicators for 10 objects	96.8	98.5	95.3
42	Random 5 indicators for 10 objects	95.4	99.4	94.8

## 4. Final remarks and future work

In this paper, we proposed the concept of logical learning through a neural network with auxiliary inputs, namely indicators, generated from the original labels, or even the original input data. The logical learning can always generate results with a higher logical accuracy that is supported by a reasoning process. We further demonstrated the robustness of our proposed logical learning in a series of simple and special indicators using the MNIST data and a set of image data from robotic grasping. A few guidelines are summarized below to help assist the design and use of our network. The proposed method provides us with a way to reflect the logical reasoning process while trying to comprehend more advanced concepts. It enables one to model the *unknown unknowns* without the loss of trust when a new and uncertain input is supplied. This process can be a meaningful one through the design of the indicators when established a prior understanding of the data is available, or a randomized one when the focus is only on a possible logic for a reasonable answer instead of the *meaning* behind.

- Any indicator is a good indicator for logical reasoning;
- Logical complexity positively relates to the confidence of an answer;
- Logical result is at the cost of overall accuracy;
- Meaningful indicators are not that good, and direct suggestions are not that bad;
- The design of the indicators is not limited by our understanding of the data.

The advancement of computing capabilities enables one to use brutal force to compute using neural networks for the most probable answer without caring much into the logic behind. The average percentage of passing logical checks in the MNIST experiments is at a relatively high level of 99.32%, leaving only a small fraction of data marked as illogical. However, this is mainly due to the high quality and simplicity of the MNIST data, which is slightly different yet consistent with our object image data from robotic grasping. It is particularly challenging when the cost of getting training data is expensive, especially when data collection requires physical interactions with the external environment, such as in robotics. When only a limited amount of data is available, it becomes necessary to utilize all aspects of the data, including the logical reasoning, physical meaning, as well as environmental parameters, etc., for a potential solution with the most trustworthiness.

There are several limitations of this study which requires further research into such auxiliary input. Although widely adopted as a benchmark in neural network research, the MNIST dataset is relatively small in size and simple in structure. This limited the design of indicators used in this paper, which are confined to cases such as a prime number or not, even or odd number, etc. Furthermore, it still requires further research into a more thorough design of the indicators to fully explore the theoretical foundations and implications of these auxiliary inputs. Last but not the least, issues such as the computation time and efficiency were not discussed in this paper, which needs to be further optimized on a general basis.

While the scope of this paper is to introduce the concept of logical learning using our network as the indicators, future work requires systematic research into the comprehensive and logical design of the indicators and our network. Another possible future work is the implementation of the proposed neural network design in robotic learning, such as object grasping. Robotic learning tasks present a realistic scenario where inputs collected from various sensors in the robotic hardware are gathered to perform an integrated task with physical interactions to the real world. This is different from most visual, audio, or text-based learning tasks where only the information is processed instead of physically interacting with the world. This difference imposes a major source of uncertainties to robotic learning, making it a potential application of logical reasoning with auxiliary inputs to improve the creditability of the learning outcomes.

In the example of detection problems such as robotic grasping tasks, a general purpose hardware platform can be setup which involves a multi-axis robotic arm, a multi-finger gripper, a tray of objects for grasping, a camera for visual sensing and a learning computer with a robotic controller for control and computation. Similar robotic platforms have been reported in various recent publications with different level of hardware complexity but usually at an expensive cost. For example, the Baxter robot with two 7-DOF arms and 2-finger parallel gripper is used by both by Pinto and Gupta (2016) and Lenz et al. (2015). Research groups at the University of California, Berkley have used various robotic systems, including a fleet of 7–14 custom-built 7-DOF arms by Google with adaptive 2-finger grippers (Levine et al., 2018), or Yumi 7-DOF arm with 2-finger parallel gripper (Mahler et al., 2017). Similar neural networks were adopted in these works (Lenz et al., 2015; Pinto and Gupta, 2016; Mahler et al., 2017; Levine et al., 2018), where the primary input will be the images taken from the tray, and the output labels will be the grasping success or failure. Specifically, in work by Levine et al. (2018), an auxiliary input is also used besides the primary input. However, they are from independent sources, both of which connect to the output labels. Thus there is no logical reasoning behind these models. Future work will focus on structurally simplify the object grasping tasks using a setup as shown in Figure 7 by mimicking the arcade claw machine (Wan and Song, 2017a) to implement the proposed network design for object grasping tasks.

## Author contributions

FW substantially contributed to the conception of the work, drafted the work for important intellectual content, approved this version to be published and accountable for all aspects of the work in ensuring that questions related to the accuracy or integrity of any part of the work are appropriately investigated and resolved. CS substantially contributed to the design of the work, critically revised the work for important intellectual content, approved this version to be published and accountable for all aspects of the work in ensuring that questions related to the accuracy or integrity of any part of the work are appropriately investigated and resolved.

### Conflict of interest statement

The authors declare that the research was conducted in the absence of any commercial or financial relationships that could be construed as a potential conflict of interest.
